# Skeletal muscle index, grip strength, and physical performance as predictors of severe chemotherapy toxicity among older adults with malignancy

**DOI:** 10.1371/journal.pone.0336968

**Published:** 2025-11-19

**Authors:** Efthymios Papadopoulos, Dmitry Rozenberg, Andy Kin On Wong, Sharon Hiu Ching Law, Sarah Costa, Angela M. Cheung, Shabbir M. H. Alibhai

**Affiliations:** 1 School of Kinesiology, Louisiana State University, Baton Rouge, Louisiana, United States of America; 2 Toronto General Hospital Research Institute, University Health Network, Toronto, Ontario, Canada; 3 Ajmera Transplant Center, University Health Network, Toronto, Ontario, Canada; 4 Division of Respirology, Department of Medicine, Temerty Faculty of Medicine, University of Toronto, Toronto, Ontario, Canada; 5 West Park Health Care Center, University Health Network, Toronto, Ontario, Canada; 6 Centre of Excellence in Skeletal Health Assessment, Joint Department of Medical Imaging, University Health Network, Toronto, Ontario, Canada; 7 Division of Epidemiology, Dalla Lana School of Public Health, University of Toronto, Toronto, Ontario, Canada; 8 Institute of Health Policy, Management and Evaluation, University of Toronto, Toronto, Ontario, Canada; 9 Department of Medicine, University Health Network, Toronto, Ontario, Canada; 10 Department of Supportive Care, Princess Margaret Cancer Centre, Toronto, Ontario, Canada; Fondazione Don Carlo Gnocchi, ITALY

## Abstract

**Background:**

Skeletal muscle index (SMI), grip strength, and physical performance have been shown to predict clinically relevant outcomes in geriatric oncology. However, their predictive ability for chemotherapy toxicity is poorly understood. We examined whether SMI, grip strength, or physical performance are independently associated with severe toxicity among older adults receiving chemotherapy.

**Methods:**

Older adults (≥65y) who had received chemotherapy at an academic cancer center between June 2015 and June 2022 were included in the analysis. SMI prior to chemotherapy was determined via computed tomography (CT), using the entire cross-sectional area of the muscle (cm^2^) at the third lumbar vertebra (L3) divided by the square of patient height in meters. Grip strength and lower extremity physical performance were measured prior to chemotherapy. Multivariable logistic regression was used to examine the independent associations between SMI, low grip strength, and low physical performance with severe (grade≥3) chemotherapy toxicity.

**Results:**

Of the 115 older adults in the study, 71.3% were males. The most common disease site was genitourinary (53.9%) and most participants received chemotherapy with palliative intent (67.8%). A total of 69 (60.0%) participants experienced at least one grade ≥3 toxicity during the study. In multivariable analyses, low grip strength per the Sarcopenia Definitions and Outcomes Consortium (SDOC) definition was significantly associated with grade ≥3 toxicity (adjusted odds ratio (OR): 2.77, 95%CI: 1.03–7.45, p = 0.044). SMI either as a continuous (OR: 1.03, 95%CI: 0.97–1.09, p = 0.40) or categorical variable (OR: 1.17, 95%CI: 0.47–2.89, p = 0.74) was not predictive of grade ≥3 toxicity. Similarly, low physical performance did not have significant associations with grade ≥3 toxicity (OR: 2.06, 95%CI: 0.86–4.95, p = 0.11).

**Conclusion:**

Low grip strength may predict grade ≥3 toxicity among older adults receiving chemotherapy. Integrating grip strength into geriatric assessment may help clinicians identify older adults who might be at greater risk for severe chemotherapy toxicity.

## Introduction

Measures of skeletal muscle mass, muscle strength, and physical performance are indicators of physical fitness and well-being and may predict clinically relevant outcomes among older adults with cancer. Several meta-analyses in oncology suggest that the risk of all-cause mortality significantly increases in the presence of low muscle mass [1], or low muscle strength [2,3], or low physical performance [2,3]. However, the relationship between skeletal muscle measures and chemotherapy outcomes is less clear due to conflicting findings.

Meta-analytic data demonstrated that patients with low CT-based muscle mass had a two-fold higher risk of dose-limiting toxicities compared to those with normal muscle mass in response to palliative chemotherapy [4]. However, these findings were not replicated by a cohort study where low CT-based muscle mass was not associated with grade 3–4 toxicity among patients (mean age: 63 years) receiving palliative chemotherapy for advanced esophagogastric cancer [5].

Regarding the relationship between grip strength with toxicity among older adults with cancer, some studies found that grip strength was significantly associated with chemotherapy toxicity [6,7] among older patients, while others failed to replicate these findings [8]. Further work demonstrated that low grip strength may predict some but not overall dose-limiting toxicity in older adults receiving chemotherapy [9]. The discrepancy in the findings of these studies may be attributed to differences in disease and treatment characteristics and covariates in multivariable analyses. Moreover, direct comparisons between grip strength and tools that have been shown to predict toxicity [10] such as the Chemotherapy Risk Assessment Scale for High-Age Patients (CRASH) [11] or Cancer Aging Research Group (CARG) [12] tools, were not examined.

The relationship between physical performance and chemotherapy outcomes is also unclear due to mixed findings. Specifically, better physical performance, as measured with the Short Physical Performance Battery (SPPB), was associated with completion of more chemotherapy cycles, fewer hospitalizations, and fewer adverse events among older adults with lung cancer [13]. However, in a study of 551 older patients with mixed cancers, SPPB was not predictive of grade ≥3 chemotherapy toxicity (SPPB <8, adjusted odds ratio (OR): 1.04, 95%CI: 0.97–1.09) [14]. The divergent findings between the two studies [13,14] may be explained by the different covariates in multivariable analyses, differences in disease characteristics, and the use of the SPPB as a continuous [13] versus a binary [14] predictor variable. Additionally, Feliu et al. [14] examined the performance of CARG in predicting severe toxicity. However, CARG and SPPB were not included in the same multivariable model, precluding direct comparisons between the two on their performance in predicting toxicity. The inconsistent findings on the role of SMI, grip strength, and physical performance in predicting chemotherapy toxicity among older adults with cancer, and the importance of improving risk stratification for geriatric patients beyond commonly used tools (e.g., CRASH) [11] and CARG [12]) underscore the need for further research in this area to optimize prediction of treatment toxicity, particularly of high-grade. Mechanistically, low muscle mass may alter the pharmacokinetics of chemotherapeutic agents, increasing toxicity [15–17]. Similarly, low grip strength and low physical performance are indices of decreased functioning, and potentially frailty [18], which has been shown to predict chemotherapy toxicity [19,20]. Grade ≥3 toxicities during chemotherapy are of clinical relevance [21] as they lead to treatment modification, as well as declines in quality of life and physical functioning and unplanned health care use, particularly among frail older patients [19]. Therefore, identifying predictors of grade ≥3 toxicity may assist clinicians with chemotherapy and supportive care planning which may optimize treatment outcomes.

The objective of this study was to examine whether skeletal muscle index (SMI), grip strength or physical performance are independently associated with severe (grade ≥3) toxicity among older adults receiving chemotherapy. We hypothesized that SMI, low grip strength, and low physical performance will each be predictive of severe chemotherapy toxicity in this cohort.

## Materials and methods

This was a retrospective cohort study that combined two groups of patients who had received chemotherapy at the Princess Margaret Cancer Centre, Toronto, Canada. The first group was comprised of older adults who had a geriatric assessment (GA) in the Older Adults with Cancer Clinic (OACC) from June 2015 to June 2022 and were subsequently treated with chemotherapy for gastrointestinal, genitourinary, gynecological cancer, or lymphoma. The second group comprised of older patients with metastatic castrate-resistant prostate cancer (mCRPC) who had received chemotherapy at the same institution and had participated in an observational study from July 2015 to April 2019 [22]. Combining the data from both cohorts aimed to increase the overall sample and statistical power. Part of the study methods are also described elsewhere [23].

The present study included participants who were ≥65 years old, were diagnosed with either gastrointestinal, genitourinary, or gynecological cancer, or lymphoma of any stage, were treated with chemotherapy at the Princess Margaret Cancer Centre, had an abdominal computed tomography (CT) scan ≤6 months prior to chemotherapy initiation, and had complete data on muscle strength and physical performance evaluated during the geriatric assessment (group 1) or as part of the baseline assessment of a research study (group 2) [24], and had complete data on anthropometric characteristics. The requirement to obtain informed consent for the present study was waived and the study was approved by the Research Ethics Board at the University Health Network (ID: 22–5600). Study data were accessed on 15/09/2022.

### Study procedures

The OACC database and participant electronic medical records were used to provide data for older adults with cancer who had received chemotherapy from June 2015 to June 2022 (group 1). The OACC database, which was created concurrently with the inception of the OACC clinic in June 2015, includes clinical information, such as disease characteristics and geriatric domains that were used for the purposes of the study. Electronic medical records were used to retrieve information on muscle strength and physical performance scores that were collected by clinical nurse specialists, routine blood markers prior to treatment, and a CT scan ≤6 months prior to chemotherapy initiation. Data of patients with mCRPC (group 2) were available from previous studies [22,24]. Patients with mCRPC had a baseline assessment prior to chemotherapy that involved collection of clinical characteristics, as well as assessment of muscle strength and physical performance by a trained research coordinator.

### Assessment of skeletal muscle mass

The cross-sectional area of the skeletal muscle was assessed at the center of the 3^rd^ lumbar vertebra (L3) using two consecutive slices (0.82 x 0.82 x 2.5 mm voxel size) of an abdominal CT scan prior to chemotherapy initiation. Segmentation and quantification of skeletal muscle followed a two-stage process. First, all images were processed through a histogram-based fully automated iterative threshold-seeking algorithm for separation of skeletal muscle from bone and adipose tissue [25] using Jupyter Notebooks (Python 3.9). Subsequently, all images were imported and reviewed on SliceOmatic software (Tomovision, Montreal, QC, Canada), and manual corrections were completed where necessary using a Hounsfield unit (HU) range between −29 to +150 for skeletal muscle [26]. The average cross-sectional area (cm^2^) between the two consecutive, manually corrected slices was divided by the patient’s square of body height in meters to derive the SMI [22].

### Assessment of muscle strength and physical performance

Muscle strength of all participants (groups 1 and 2) was assessed via grip strength using standard procedures [27]. In brief, participants from the seated position performed two attempts in the dominant hand using a Jamar dynamometer (Sammons Preston, Bolingbrook, IL, USA) and the higher of the two attempts was used in kilograms. Low grip strength was defined per the Sarcopenia Definitions and Outcomes Consortium (SDOC) criteria (<35.5 kg for men and <20 kg for women) [28]. Physical performance of older adults in the OACC (group 1) was assessed using the Short Physical Performance Battery [29], whereas the 4-meter gait speed was used for older men with mCPRC (group 2) [27]. The cutoff to define low physical performance via the SPPB and the 4-meter gait speed were ≤8/12 [30], and <0.8m/s [28], respectively.

### Study outcomes

The occurrence of severe (grade ≥3) toxicity was assessed from chemotherapy initiation until discontinuation or loss to follow up until June 2022 using the Common Terminology Criteria for Adverse Events (CTCAE) version 4.0. For older adults who had undergone a GA at the OACC, severe toxicities (hematologic and non-hematologic) were captured via routine clinical care visits recorded in electronic medical records. For older men with mCRPC, non-hematologic grade ≥3 toxicities were captured every 3 weeks during the main study, while hematologic grade ≥3 toxicities were retrieved from electronic medical records.

### Statistical analysis

The characteristics of study participants at baseline were summarized using means and standard deviations for continuous variables and frequencies and proportions for categorical data. Multivariable logistic regression models were developed to assess the independent associations of SMI, low grip strength, and low physical performance with severe (grade ≥ 3) toxicity. Covariates in multivariable analyses included variables with a p value <0.10 in the univariate analysis. The predictor variables (SMI, low grip strength, and low physical performance) were forced into the same multivariable models regardless of their p value in the univariate analysis. SMI was treated as a continuous variable in the main analysis to better understand its relationship with the occurrence of grade ≥ 3 toxicity. In a sensitivity analysis, SMI was dichotomized using sex- and body mass index-based cutoffs per Martin et al. [31]. Specifically, for men with a body mass index (BMI) <25, low SMI was defined as <43 cm^2^/m^2^, while for men with a BMI ≥ 25, the cutoff for low SMI was < 53 cm^2^/m^2^ [31]. For women, the cutoff for low SMI was < 41 cm^2^/m^2^ [31]. A sensitivity analysis was conducted to examine whether low grip strength per the European Working Group on Sarcopenia in Older People 2 (EWGSOP2) criteria (<27 kg for males and <16 kg for females) [30] were predictive of any grade ≥ 3 toxicity. This approach was taken as it is currently unclear which grip strength cutoffs are associated with severe chemotherapy toxicity among older adults with cancer. A p-value of <0.05 was used to define statistical significance. All analyses were performed using IBM SPSS for Windows, Version 29.0. Armonk, NY, USA: IBM Corp.

### Results

A total of 115 older adults (mean age: 77.1 years; range 65–91 years) were included in the analysis (Table 1). Reasons for exclusion are listed in S1 Fig. Most participants were males (71.3%), had genitourinary disease (53.9%) and were undergoing palliative chemotherapy (67.8%). Additionally, the majority of participants had metastatic disease (63.5%). Low SMI (72.2%) and low grip strength (76.5%) were highly prevalent, whereas 40% of participants had low physical performance (Table 1). The characteristics of study participants by cohort and by toxicity status are shown in S1 and S2 Tables, respectively.

**Table 1:** Characteristics of study participants at baseline (n = 115).

Variable	Mean (SD) or n (%)
Age (years), mean (SD)	77.1 (6.6) (range: 65–91)
Sex, n (%)	
Males	82 (71.3)
Treatment intent, n (%)	
Palliative	78 (67.8)
Disease site, n (%)	
Genitourinary	62 (53.9)
Gastrointestinal	17 (14.8)
Gynecological	15 (13.0)
Lymphoma	21 (18.3)
Disease stage, n (%)	
Localized	11 (9.6)
Locally advanced	10 (8.7)
Hematologic	21 (18.3)
Metastatic	73 (63.5)
Chemotherapy agent(s)	
Alkylating	14 (12.2)
Alkylating & antimetabolites	13 (11.3)
Alkylating & monoclonal antibodies	17 (14.8)
Alkylating & taxanes	8 (7.0)
Antimetabolites	7 (6.1)
Antimetabolites & taxanes	2 (1.7)
Antimetabolites & monoclonal antibody	1 (0.9)
Taxanes	53 (46.1)
Chemotherapy duration (days), mean, SD	108.3 (75.1)
Body mass index, mean (SD)	26.8 (5.1)
Dependent on one or more IADLs, n (%)	61 (53.0)
Cognitive impairment, n (%)	48 (41.7)
Albumin (g/L), mean (SD)^a^	38.5 (3.3)
Alkaline phosphatase (u/L), Median (IQR)	91.50 (69.3-133.8)
Hemoglobin (g/L), mean (SD)	116.3 (18.9)
Lactate dehydrogenase (u/L), mean (SD)	273.4 (104.7)
Neutrophil-to-lymphocyte ratio (median: ≥ 3.7), n (%)	58 (50.4)
Grip strength (kg), mean (SD)	25.9 (8.1)
Low Grip strength per SDOC, n (%)	88 (76.5)
Low physical performance n (%)^b^	46 (40.0)
SPPB total score, mean (SD)	8.8 (2.7)
4-meter gait speed m/s, mean (SD)	0.8 (0.22)
Days between CT and chemotherapy start, median (IQR)	34 (17 - 60)
SMI (cm^2^/m^2^), mean (SD)	41.4 (7.2)
Low SMI, n (%)	83 (72.2)

CT: computed tomography; IADLs = instrumental activities of daily living; IQR: interquartile range; SDOC = Sarcopenia Definitions and Outcomes Consortium; SMI = skeletal muscle index; SPPB = Short Physical Performance Battery.

^a^ n = 23 (20%) of participants did not have an available albumin value.

^b^ Physical performance among the 68 older adults who had a geriatric assessment at the OACC clinic was assessed using the SPPB. For the 47 men with mCRPC, physical performance was assessed using the 4-meter gait speed.

Note: Cognitive function of older patients in the OACC (group 1) was assessed by a clinical nurse specialist via the Mini-Cog [32], and a score of <4/5 was used to identify cognitive impairment. Cognitive function of older patients with mCRPC (group 2) was assessed by a research coordinator via the Montreal Cognitive Assessment using a cutoff of <25 to identify cognitive impairment [33].

### Risk of grade ≥3 toxicity

**Fig 1:**
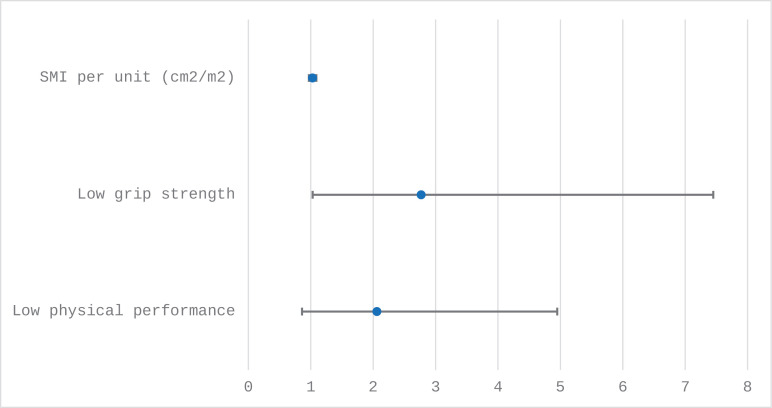
Adjusted odds ratios of skeletal muscle measures as predictors of grade ≥ 3 toxicity among older adults receiving chemotherapy.

A total of 69 (60%) participants experienced a grade ≥3 toxicity during chemotherapy. The number and type of all grade ≥3 toxicities are listed in S3 Table. In multivariable analysis, males had a significantly higher risk of grade ≥3 toxicity compared to females (adjusted odds ratio (aOR): 2.87, 95%CI: 1.10–7,49, p < 0.001) (Table 2). SMI was not associated with the occurrence of grade ≥3 toxicity (aOR: 1.03, 95%CI: 0.97–1.09, p = 0.40). However, low grip strength per the SDOC criteria was an independent predictor of grade ≥3 toxicity (aOR: 2.77, 95%CI: 1.03–7.45, p = 0.044) (Table 2 and Fig 1).

**Table 2:** Associations between skeletal muscle measures and grade ≥3 toxicity.

Variable	Unadjusted OR (95%CI)	*p*	Adjusted OR (95%CI)^a^ (n = 114)	*p*
Age per decade	1.71 (0.95-3.09)	0.074	1.89 (0.98-3.67)	0.058
Sex (males)	2.77 (1.21-6.34)	0.016	2.87 (1.10-7.49)	0.031
Body mass index per kg/m^2^	1.05 (0.97-1.13)	0.20		
Genitourinary cancer	1.51 (0.71-3.19)	0.29		
Treatment intent (palliative)	1.69 (0.77-3.74)	0.19		
Disease stage				
Localized or locally advanced	1.01 (0.37-2.75)	0.98		
Hematologic	0.68 (0.26-1.82)	0.45		
Metastatic	ref.		ref.	
Cognitive impairment^b^	1.95 (0.89-4.25)	0.093	1.66 (0.71-3.88)	0.24
Dependence in ≥1 IADLs	1.42 (0.67-3.00)	0.36		
Neutrophil-to-lymphocyte ratio (continuous)	0.98 (0.89-1.08)	0.74		
Lactate dehydrogenase (IU/L)	1.00 (0.99-1.01)	0.38		
Alkaline phosphatase (IU/L)	1.29 (0.71-2.37)	0.41		
Hemoglobin per 10-unit decrease (g/L)	1.16 (0.95-1.43)	0.15		
Low grip strength per SDOC	3.46 (1.41-8.50)	0.007	2.77 (1.03-7.45)	0.044
SMI per unit (cm^2^/m^2^)	1.04 (0.98-1.10)	0.16	1.03 (0.97-1.09)	0.40
Low physical performance	1.98 (0.90-4.34)	0.089	2.06 (0.86-4.95)	0.11

^a^ Hosmer-Lemeshow test = 4.24, p = 0.83; c-stat: 0.74.

^b^ One participant had missing information on cognitive function.

IADLs = instrumental activities of daily living; SDOC = Sarcopenia Definitions and Outcomes Consortium; SMI = skeletal muscle index.

Note: The multivariable analysis included variables with a p value <0.10 in the univariate analysis, in addition to the main predictors (low grip strength, SMI (cm^2^/m^2^), and low physical performance).

A non-significant association was found between low physical performance and grade ≥3 toxicity (OR: 2.06, 95%CI: 0.86–4.95, p = 0.11) (Table 2). The multivariable model had good calibration (HL p = 0.83) and AUC (c-statistic: 0.74). (Table 2).

### Sensitivity analysis with skeletal muscle index as a categorical variable

S4 Table lists the sensitivity analysis that explored the associations between low SMI and grade ≥3 toxicity. Patients with low SMI had a non-significant 17% higher risk of grade ≥3 toxicity (aOR: 1.17, 95%CI: 0.47–2.89, p = 0.74) (S4 Table). In line with the primary analysis, low grip strength per the SDOC criteria was a significant predictor of grade ≥3 toxicity (aOR: 2.70, 95%CI: 1.01–7.22, p = 0.048), while the association between low physical performance and grade ≥3 toxicity did not reach statistical significance (aOR: 2.03, 95%CI: 0.84–4.88, p = 0.11).

### Sensitivity analysis with low grip strength per the EWGSOP2 criteria

Table 3 lists the sensitivity analysis that examined the associations between low grip strength using the EWGSOP2 criteria. The associations between low grip strength and grade ≥3 toxicity was no longer significant after using the EWGSOP2 criteria (aOR: 1.76, 95%CI: 0.71–4.38, p = 0.23). (Table 3).

**Table 3:** Sensitivity analysis of the associations between skeletal muscle measures and grade ≥3 toxicity with low grip strength per the EWGSOP2 criteria.

Variable	Adjusted OR (95%CI) (n = 114)^a^	*p*
Age per decade	2.05 (1.06-3.94)	0.033
Sex (males)	3.45 (1.34-8.86)	0.010
Cognitive impairment^b^	1.72 (0.75-3.97)	0.20
Low grip strength per EWGSOP2	1.76 (0.71-4.38)	0.23
SMI per unit (cm^2^/m^2^)	1.02 (0.96-1.08)	0.58
Low physical performance	2.10 (0.87-5.08)	0.099

^a^ Hosmer-Lemeshow test = 12.5, p = 0.13; c-stat: 0.72.

^^b^^ One participant had missing information on cognitive function.

EWGSOP2 = European Working Group on Sarcopenia in Older People 2; SMI = skeletal muscle index.

## Discussion

This study sought to test whether SMI, low grip strength, or low physical performance are associated with grade ≥3 toxicity among older adults with cancer undergoing chemotherapy. Our findings suggest that of the three predictor variables (SMI, low grip strength, and low physical performance), only low grip strength per the SDOC criteria had the best performance and retained statistical significance in multivariable analyses.

SMI was first examined as a continuous variable which may be more appropriate for research purposes instead of dichotomizing participants, that inherently, assumes the same risk for those above or below a threshold [16]. However, thresholds are more meaningful for clinicians to identify patients with low SMI. Thus, in a sensitivity analysis, we used sex- and BMI-based cutoffs [31], which have previously been shown to predict grade ≥3 toxicity among younger groups of patients (median age: 61 years) undergoing chemoradiation for locally advanced esophageal cancer [34] as well as adults (median age: 53 years) receiving chemotherapy for peritoneal carcinomatosis from colorectal cancer [35]. Nonetheless, in our study, participants with low SMI did not have a significantly higher risk of grade ≥3 toxicity compared to those with normal SMI. Our findings align with those reported by Dijksterhuis et al [5] where baseline SMI either as a continuous or categorical variable based on the criteria by Martin et al [31], was not significantly associated with grade ≥3 toxicity among patients undergoing palliative chemotherapy [5]. The discrepancy between our findings and prior literature may be attributed to differences in disease- and treatment-related characteristics of study participants. For example, 41% of study participants were men with mCRPC who had undergone ADT prior to chemotherapy, which might have led to changes in fat and muscle composition. It is also possible that skeletal muscle density may provide additional information on risk stratification compared to SMI. In a study of 145 patients (mean age: 59.0 years) undergoing chemoimmunotherapy for lymphoma, muscle density but not SMI was associated with significantly better treatment response rates [36] per Cheson et al. [37].

Low grip strength per the SDOC criteria was the only significant predictor of severe chemotherapy toxicity in this cohort. Specifically, participants with low grip strength were approximately 2.7 times more likely to experience grade ≥3 toxicity. Previous work has also demonstrated associations between grip strength and chemotherapy toxicity in older patients [6,7], but others failed to corroborate this notion [8]. The divergent findings among studies may be explained, in part, due to the use of different grip strength cut points. Notably, defining low grip strength per the EWGSOP2 criteria in a sensitivity analysis resulted in a non-significant association between low grip strength and grade ≥3 toxicity. It is currently unclear which grip strength cutoffs should be used to define muscle weakness in geriatric oncology. Although the discriminative ability of common grip strength cutoffs for abnormal GA has recently been examined [38], data on chemotherapy toxicity in relation to different grip strength criteria are scarce. However, our results are clinically relevant and suggest that low grip strength using the SDOC cut points [28] appear to be predictive of severe chemotherapy toxicity among older adults with cancer. Outcome-specific (e.g., chemotherapy toxicity) cutoffs for low grip strength from larger studies are needed to aid risk stratification among older adults with cancer. Grip strength can easily be assessed during GA [39] within the domain of physical function/performance to further characterize the older adult’s muscle function prior to chemotherapy initiation. For older adults with low CARG and low grip strength, more caution with upfront dose reduction and/or close monitoring during the initial cycles of chemotherapy is advised. However, in the absence of CARG, oncologists should consider reducing the treatment intensity for an older adult with low grip strength. From a mechanistic perspective, low grip strength as a measure of global muscle strength [40] may be a better indicator of frailty than SMI alone. Indeed, muscle strength is included in the frailty criteria as described by Fried et al. [18], while it is well established that frailty is an important prognostic marker of chemotherapy toxicity [19,20], which may explain our findings.

Low physical performance which is also indicative of frailty [41] was associated with a non-significant two-fold risk of grade ≥3 toxicity. The lack of statistical significance between low physical performance and severe toxicity is likely attributed to the small sample size and inadequate power given the observed odds ratio and 95% confidence intervals in multivariable analyses. Another plausible explanation for this non-significant association is the reliance on different physical performance tests among study participants (SPPB and 4-meter gait speed). Whether low physical performance is associated with severe toxicity among older adults during chemotherapy requires further research with larger studies due to the heterogeneity in findings [13,14].

An important strength of this study is the exclusive inclusion of older adults, an underrepresented population in clinical oncology research [42]. Additionally, the assessment of grade ≥3 toxicity included both hematological and non-hematological severe toxicities. Limitations include the small sample size, exclusion of albumin from the analysis due to missing data, cohort heterogeneity, variability in time of exposure to treatment, as well as lack of information on dose reduction and treatment delays which might have influenced the associations between the predictor variables and grade ≥3 toxicity. Moreover, we were unable to examine physical performance as a continuous variable given that it was heterogeneously assessed using either the SPPB or the 4-meter gait speed, as well as the lack of component scores for SPPB. Additionally, the lack of physical activity data is another limitation as physically active patients may have a lower risk of chemotherapy toxicities such as fatigue [43] and pain [44].

Collectively, grip strength and physical performance can be assessed during GA and during follow-up to provide information on the patient’s physical fitness and inform targeted interventions (e.g., exercise). Regarding SMI, semi-automated approaches of muscle segmentation via CT require additional time and training for clinicians. However, fully-automated approaches for quantifying body composition may overcome these barriers [45], allowing clinicians to obtain information on patient’s SMI. Alternatively, clinicians may assess lean soft tissue mass (i.e., fat- and bone-mineral-free mass) using dual x-ray absorptiometry, or fat-free mass using bioelectrical impedance analysis (BIA) [46]. However, whether these measures can predict the risk of toxicity should be further examined through validation studies in older adults with cancer [46].

Given the scarcity of evidence and mixed findings of existing studies on this topic, future research from large studies is needed to assess the performance of grip strength, SMI, and physical performance in predicting grade ≥3 toxicities in geriatric oncology. Additionally, future studies should examine whether the associations between measures of muscle mass, function, and chemotherapy toxicity differ between older adults with solid versus hematological malignancies, and among different chemotherapeutic agents. Another area of future research pertains to the identification of outcome-specific cutoffs for risk stratification. For example, the cutoffs for low grip strength used in this study were derived by different expert groups on sarcopenia but none of these cutoffs were based on cohorts of older adults with cancer [28,30]. Similarly, the sex- and BMI-based cutoffs for SMI were originally developed to assess mortality among patients with solid malignancies [31]. Large studies should identify toxicity-based cutoffs for low grip strength and SMI among older adults with cancer undergoing chemotherapy. Finally, in prostate cancer specifically, studies should also examine whether the predictive value of grip strength, SMI, and physical performance measures on chemotherapy toxicity are modified by duration of prior androgen deprivation use.

### Conclusion

Among measures of skeletal muscle mass, strength, and physical performance, only low grip strength per the SDOC definition was an independent predictor of grade ≥3 toxicity among older adults with cancer undergoing chemotherapy. Grip strength during GA may inform personalized treatment plans aimed at minimizing severe toxicity during chemotherapy. Additional studies with adequate power are needed to confirm the role of grip strength, SMI, and physical performance in predicting severe chemotherapy toxicity among older adults with cancer.

## Supporting information

S1 Fig Participant Flow Chart. (DOCX)

S1 Table Characteristics of study participants by cohort. (DOCX)

S2 Table Characteristics of study participants by toxicity status. (DOCX)

S3 Table Grade ≥3 hematologic and non-hematologic toxicities during chemotherapy. (DOCX)

S4 Table Sensitivity analysis of the associations between skeletal muscle measures and grade ≥3 toxicity with SMI as a categorical variable. (DOCX)
