# Supplementary material for: Skeletal muscle index, grip strength, and physical performance as predictors of severe chemotherapy toxicity among older adults with malignancy
Source: PLoS One. 2025 Nov 19;20(11):e0336968. doi: 10.1371/journal.pone.0336968 (PMC12629486; doi:10.1371/journal.pone.0336968)
Supplement: S1 Fig — (DOCX) [file pone.0336968.s001.docx]

**S1 Figure.** Participant Flow Chart

Older patients with mCRPC prior to chemotherapy who had participated in the observational study^a^ (n=47)

Older adults who were seen in the OACC prior to chemotherapy from June 2015 to June 2022 (n=177)

**Excluded:**

- (n=78) Did not have an abdominal CT scan ≤6 prior to chemotherapy initiation
- (n=8) had started chemotherapy prior to GA
- (n=7) did not have chemotherapy after GA
- (n=4) did not have available clinical notes after GA
- (n=3) had missing muscle strength and/or physical performance
- (n=3) issues with CT scan (e.g., errors not showing full scan, incorrect volume)
- (n=3) participated in observational study
- (n=2) missing anthropometric characteristics
- (n=1) had chemotherapy elsewhere

N=115 included in the analysis

^a^n=47 participants had participated in an observational study and were included in the present analysis to increase the sample size and statistical power.

OACC= Older Adults with Cancer Clinic
