# Supplementary material for: Skeletal muscle index, grip strength, and physical performance as predictors of severe chemotherapy toxicity among older adults with malignancy
Source: PLoS One. 2025 Nov 19;20(11):e0336968. doi: 10.1371/journal.pone.0336968 (PMC12629486; doi:10.1371/journal.pone.0336968)
Supplement: S1 Table — (DOCX) [file pone.0336968.s002.docx]

**S1 Table.** Characteristics of study participants by cohort (n=115)

| **Variable** | **OACC patients (group 1)**  **n=68** | **Men with mCRPC (group 2)**  **n=47** |
| --- | --- | --- |
| Age (years), mean (SD) | 79.2 (5.7) | 74.2 (6.7) |
| Sex, n (%) |  |  |
| Males | 35 (51.5) | 47 (100.0) |
| Treatment intent, n (%) |  |  |
| Palliative | 31 (45.6) | 47 (100.0) |
| Disease site, n (%) |  |  |
| Genitourinary | 15 (22.1) | 47 (100.0) |
| Gastrointestinal | 17 (25.0) | 0 (0) |
| Gynecological | 15 (22.1) | 0 (0) |
| Lymphoma | 21 (30.9) | 0 (0) |
| Disease stage, n (%) |  |  |
| Localized | 11 (16.2) | (0) |
| Locally advanced | 10 (14.7) | (0) |
| Hematologic | 21 (30.9) | (0) |
| Metastatic | 26 (38.2) | 47 (100) |
| Chemotherapy agent(s) |  |  |
| Alkylating | 14 (20.6) | 0 (0) |
| Alkylating & antimetabolites | 13 (19.1) | 0 (0) |
| Alkylating & monoclonal antibodies | 17 (25.0) | 0 (0) |
| Alkylating & taxanes | 8 (11.8) | 0 (0) |
| Antimetabolites | 7 (10.3) | 0 (0) |
| Antimetabolites & taxanes | 2 (2.9) | 0 (0) |
| Antimetabolites & monoclonal antibody | 1 (1.5) | 0 (0) |
| Taxanes | 6 (8.8) | 47 (100.0) |
| Body mass index, mean (SD) | 25.8 (4.7) | 28.2 (5.2) |
| Dependent in one or more IADLs, n (%) | 36 (52.9) | 25 (53.2) |
| Cognitive impairment, n (%) | 21 (30.9) | 27 (57.4) |
| Albumin (g/L), mean (SD)^a^ | 38.5 (3.2) | 38.5 (3.4) |
| Alkaline phosphatase (u/L), Median (IQR) | 79.5 (65.0-105.0) | 110.0 (84.0-239.2) |
| Hemoglobin (g/L), mean (SD) | 113.2 (20.1) | 120.8 (16.3) |
| Lactate dehydrogenase (u/L), mean (SD) | 256.9 (93.4) | 297.7 (116.4) |
| Neutrophil-to-lymphocyte ratio, mean (SD) | 4.8 (4.5) | 4.4 (2.5) |
| Grip strength (kg), mean (SD) | 23.4 (7.9) | 29.6 (6.8) |
| Low Grip strength per SDOC, n (%) | 50 (73.5) | 38 (80.9) |
| Low physical performance n (%)^b^ | 24 (35.3) | 22 (46.8) |
| SPPB total score, mean (SD) | 8.9 (2.7) | N/A |
| 4-meter gait speed m/s, mean (SD) | N/A | 0.9 (0.2) |
| SMI (cm^2^/m^2^), mean (SD) | 41.7 (7.1) | 40.9 (7.3) |
| Low SMI, n (%) | 44 (64.7) | 39 (83.0) |

IADLs= instrumental activities of daily living; IQR: interquartile range; SDOC= Sarcopenia Definitions and Outcomes Consortium; SMI= skeletal muscle index; SPPB= Short Physical Performance Battery
