# Supplementary material for: Skeletal muscle index, grip strength, and physical performance as predictors of severe chemotherapy toxicity among older adults with malignancy
Source: PLoS One. 2025 Nov 19;20(11):e0336968. doi: 10.1371/journal.pone.0336968 (PMC12629486; doi:10.1371/journal.pone.0336968)
Supplement: S2 Table — (DOCX) [file pone.0336968.s003.docx]

**S2 Table.** Characteristics of study participants by toxicity status (n=115)

| **Variable** | **Participants without grade ≥3 toxicity**  **(n= 46)** | **Participants with grade ≥3 toxicity**  **(n= 69)** |
| --- | --- | --- |
| Age (years), mean (SD) | 75.8 (6.3) | 78.0 (6.6) |
| Sex, n (%) |  |  |
| Males | 27 (58.7) | 55 (79.7) |
| Treatment intent, n (%) |  |  |
| Palliative | 28 (60.9) | 50 (72.5) |
| Disease site, n (%) |  |  |
| Genitourinary | 22 (47.8) | 40 (58.0) |
| Gastrointestinal | 7 (15.2) | 10 (14.5) |
| Gynecological | 7 (15.2) | 8 (11.6) |
| Lymphoma | 10 (21.7) | 11 (15.9) |
| Disease stage, n (%) |  |  |
| Localized | 6 (13.0) | 5 (7.2) |
| Locally advanced | 2 (4.3) | 8 (11.6) |
| Hematologic | 10 (21.7) | 11 (15.9) |
| Metastatic | 28 (60.9) | 45 (65.2) |
| Chemotherapy agent(s) |  |  |
| Alkylating | 5 (10.9) | 9 (13.0) |
| Alkylating & antimetabolites | 3 (6.5) | 10 (14.5) |
| Alkylating & monoclonal antibodies | 9 (19.6) | 8 (11.6) |
| Alkylating & taxanes | 4 (8.7) | 4 (5.8) |
| Antimetabolites | 3 (6.5) | 4 (5.8) |
| Antimetabolites & taxanes | 1 (2.2) | 1 (1.4) |
| Antimetabolites & monoclonal antibody | 9 (19.6) | 1 (1.4) |
| Taxanes | 21 (45.7) | 32 (46.4) |
| Body mass index, mean (SD) | 26.0 (4.9) | 27.2 (5.1) |
| Dependent in one or more IADLs, n (%) | 22 (47.8) | 39 (56.5) |
| Cognitive impairment, n (%) | 15 (32.6) | 33 (47.8) |
| Albumin (g/L), mean (SD)^a^ | 39.7 (3.2) | 37.8 (3.1) |
| Alkaline phosphatase (u/L), Median (IQR) | 128.2 (70.0-116.0) | 147.2 (66.0-144.2) |
| Hemoglobin (g/L), mean (SD) | 119.4 (20.5) | 114.1 (17.7) |
| Lactate dehydrogenase (u/L), mean (SD) | 262.8 (85.8) | 280.5 (115.9) |
| Neutrophil-to-lymphocyte ratio, mean (SD) | 4.7 (3.7) | 4.5 (3.9) |
| Grip strength (kg), mean (SD) | 26.2 (8.1) | 25.7 (8.2) |
| Low Grip strength per SDOC, n (%) | 29 (63.0) | 59 (85.5) |
| Low physical performance n (%)^b^ | 14 (30.4) | 32 (46.4) |
| SPPB total score, mean (SD) | 9.7 (2.4) | 8.3 (2.9) |
| 4-meter gait speed m/s, mean (SD) | 0.8 (0.1) | 0.8 (0.2) |
| SMI (cm^2^/m^2^), mean (SD) | 40.5 (7.1) | 41.9 (7.2) |
| Low SMI, n (%) | 32 (69.6) | 51 (73.9) |

IADLs= instrumental activities of daily living; IQR: interquartile range; SDOC= Sarcopenia Definitions and Outcomes Consortium; SMI= skeletal muscle index; SPPB= Short Physical Performance Battery
