# Supplementary material for: Skeletal muscle index, grip strength, and physical performance as predictors of severe chemotherapy toxicity among older adults with malignancy
Source: PLoS One. 2025 Nov 19;20(11):e0336968. doi: 10.1371/journal.pone.0336968 (PMC12629486; doi:10.1371/journal.pone.0336968)
Supplement: S3 Table — (DOCX) [file pone.0336968.s004.docx]

**S3 Table**. Grade ≥3 hematologic and non-hematologic toxicities during chemotherapy.

| **Grade ≥3 hematologic toxicity** | Number of participants who experienced the respective toxicity at least once, n (%) |
| --- | --- |
| Any grade ≥3 hematologic* | 34 (30.0) |
| Anemia | 14 (12.2) |
| Neutropenia | 24 (20.9) |
| Thrombocytopenia | 1 (0.9) |
| **Grade ≥3 non-hematologic toxicity** | Number of participants who experienced the respective toxicity at least once, n (%) |
| Any grade ≥3 non-hematologic* | 58 (50.4) |
| Anxiety | 1 (0.9) |
| ALP increased | 1 (0.9) |
| ALT increased | 1 (0.9) |
| Arthritis | 1 (0.9) |
| AST increased | 1 (0.9) |
| Atrial fibrillation | 1 (0.9) |
| Confusion | 1 (0.9) |
| Dehydration | 3 (2.6) |
| Delirium | 1 (0.9) |
| Diarrhea | 1 (0.9) |
| Dizziness | 1 (0.9) |
| Dyspnea | 2 (1.7) |
| Falls | 4 (3.5) |
| Fatigue | 15 (13.0) |
| Fever | 1 (0.9) |
| Gastric obstruction | 1 (0.9) |
| Hematuria | 1 (0.9) |
| High Creatinine | 2 (1.7) |
| Hip fracture | 1 (0.9) |
| Hydronephrosis | 2 (1.7) |
| Hyperglycemia | 2 (1.7) |
| Hyperkalemia | 1 (0.9) |
| Hypophosphatemia | 5 (4.3) |
| Hyponatremia | 7 (6.1) |
| Hypocalcemia | 2 (1.7) |
| Hypokalemia | 2 (1.7) |
| Hypertension | 4 (3.5) |
| Hypotension | 3 (2.6) |
| Infection | 5 (4.3) |
| Insomnia | 1 (0.9) |
| Muscle weakness | 1 (0.9) |
| Myocardial infarction | 1 (0.9) |
| Pain | 4 (3.5) |
| Periodontal disease | 1 (0.9) |
| Pneumonitis | 1 (0.9) |
| Rash | 1 (0.9) |
| Seizure | 1 (0.9) |
| Sepsis | 2 (1.7) |
| Sore throat | 1 (0.9) |
| Syncope | 3 (2.6) |
| Thromboembolism | 5 (4.3) |
| Urinary obstruction | 1 (0.9) |
| Weight loss | 1 (0.9) |

Note: *Some participants experienced more than one grade ≥3 toxicity. Grade ≥3 hematologic toxicities occurred in 34 (29.6%) participants. Grade ≥3 non-hematologic toxicities occurred in 58 (50.4%) participants. A total of 69 (60%) participants had at least one grade ≥3 toxicity (study outcome).
