# Supplementary material for: Skeletal muscle index, grip strength, and physical performance as predictors of severe chemotherapy toxicity among older adults with malignancy
Source: PLoS One. 2025 Nov 19;20(11):e0336968. doi: 10.1371/journal.pone.0336968 (PMC12629486; doi:10.1371/journal.pone.0336968)
Supplement: S4 Table — (DOCX) [file pone.0336968.s005.docx]

**S4 Table**. Sensitivity analysis of the associations between skeletal muscle measures and grade ≥3 toxicity with SMI as a categorical variable

| **Variable** | **Adjusted OR (95%CI) (n=114)^a^** | ***p*** |
| --- | --- | --- |
| Age per decade | 1.89 (0.98-3.67) | 0.057 |
| Sex (males) | 3.09 (1.21-7.91) | 0.019 |
| Cognitive impairment^b^ | 1.66 (0.71-3.87) | 0.24 |
| Low grip strength per SDOC | 2.70 (1.01-7.22) | 0.048 |
| Low SMI | 1.17 (0.47-2.89) | 0.74 |
| Low physical performance | 2.03 (0.84-4.88) | 0.11 |

^a^Hosmer-Lemeshow test= 4.58, p= 0.80; c-stat: 0.74

^b^One participant had missing information on cognitive function

SDOC= Sarcopenia Definitions and Outcomes Consortium; SMI= skeletal muscle index
